# Analysis and reporting of stepped wedge randomised controlled trials: synthesis and critical appraisal of published studies, 2010 to 2014

**DOI:** 10.1186/s13063-015-0838-3

**Published:** 2015-08-17

**Authors:** Calum Davey, James Hargreaves, Jennifer A Thompson, Andrew J Copas, Emma Beard, James J Lewis, Katherine L Fielding

**Affiliations:** Department of Social and Environmental Health Research, London School of Hygiene and Tropical Medicine, London, UK; Department of Infectious Disease Epidemiology, London School of Hygiene and Tropical Medicine, London, UK; London Hub for Trials Methodology Research, MRC Clinical Trials Unit at University College London, London, UK; MRC Clinical Trials Unit at University College London, London, UK; Department of Clinical, Educational and Health Psychology, University College London, London, UK; Department of Epidemiology and Public Health, University College London, London, UK; MRC Tropical Epidemiology Group, Department of Infectious Disease Epidemiology, London School of Hygiene and Tropical Medicine, London, UK

**Keywords:** stepped wedge trials, analysis, methodology, public health

## Abstract

**Background:**

Stepped wedge cluster randomised trials introduce interventions to groups of clusters in a random order and have been used to evaluate interventions for health and wellbeing. Standardised guidance for reporting stepped wedge trials is currently absent, and a range of potential analytic approaches have been described.

**Methods:**

We systematically identified and reviewed recently published (2010 to 2014) analyses of stepped wedge trials. We extracted data and described the range of reporting and analysis approaches taken across all studies. We critically appraised the strategy described by three trials chosen to reflect a range of design characteristics.

**Results:**

Ten reports of completed analyses were identified. Reporting varied: seven of the studies included a CONSORT diagram, and only five also included a diagram of the intervention rollout. Seven assessed the balance achieved by randomisation, and there was considerable heterogeneity among the approaches used. Only six reported the trend in the outcome over time. All used both ‘horizontal’ and ‘vertical’ information to estimate the intervention effect: eight adjusted for time with a fixed effect, one used time as a condition using a Cox proportional hazards model, and one did not account for time trends. The majority used simple random effects to account for clustering and repeat measures, assuming a common intervention effect across clusters. Outcome data from before and after the rollout period were often included in the primary analysis. Potential lags in the outcome response to the intervention were rarely investigated. We use three case studies to illustrate different approaches to analysis and reporting.

**Conclusions:**

There is considerable heterogeneity in the reporting of stepped wedge cluster randomised trials. Correct specification of the time-trend underlies the validity of the analytical approaches. The possibility that intervention effects vary by cluster or over time should be considered. Further work should be done to standardise the reporting of the design, attrition, balance, and time-trends in stepped wedge trials.

## Background

The stepped wedge cluster randomised controlled trial design (SWT) has been used to evaluate interventions to improve health, as well as other aspects of wellbeing [1]. Although SWTs are increasingly used [2], unlike cluster randomised controlled trials (CRT), no standards exist for reporting or for analysis.

The reporting and analysis of SWTs pose many of the same challenges as for CRTs, and the guiding principles developed for CRTs can be applied. However some challenges are unique to SWTs, and guidance to overcome them is currently absent. One issue is standardised reporting of the design of SWTs, and Copas *et al.*, in this series, addresses terminology and a taxonomy of stepped-wedge trials for clearer presentation of the designs [3]. In this article we focus on two further issues: reporting of results of SWTs, and selecting an optimal analysis strategy that is statistically efficient and leads to unbiased estimates of the effect of the intervention with appropriately characterised confidence levels.

We first discuss the two issues outlined above in more detail. We then describe how ten recently reported SWTs approached these two issues. Finally, we critically appraise the analytic approach taken by three ‘case studies’ that represent a range of different elements of SWT design. We conclude by discussing issues raised by this investigation and identify some potential ways forward.

### Issues in the reporting and analysis of an SWT

Aspects of the design of SWTs are described in detail in Copas *et al*. [3]. Clusters are collections of individuals, such as schools, homes, or hospitals. SWTs randomly allocate clusters to ‘groups’ of clusters that cross over the intervention at different ‘crossover points’. SWTs have up to three main phases [3]. For all SWTs there will be a ‘rollout period’ during which time groups of clusters are crossing over from the control condition (often ‘business as usual’) to the intervention condition [4]. At any one time during this rollout period, some groups of clusters will have been allocated to be receiving the intervention condition while others will have been allocated to be receiving the control condition. The time period between the crossover of successive groups is referred to here as the time between successive crossover points, and sometimes elsewhere as ‘step length’. Outcome data may be collected before the rollout period, when all clusters are in the control condition, or later, when all clusters are in the intervention condition.

SWTs are characterised by the timing of the participants’ enrolment and exposure to control and/or intervention conditions within the trial, the duration of follow-up, and the measurements collected during follow-up. For example, individuals may be enrolled and outcome data may be collected by following individuals over time until some event occurs, such as death or becoming a disease case, or until any other ‘censoring’ event when they become ineligible for follow-up. Alternatively, data may be collected at discrete time points over the course of follow-up. Analysing and reporting the data arising from this array of study design characteristics pose some common and some unique challenges.

### Reporting of SWTs

Standardisation of reporting practices has greatly aided the interpretation and synthesis of results from CRTs [5]. In contrast, there is no standard reporting template for SWTs in public health or any other field.

Two features make SWTs more complex to report than the equivalent CRTs, requiring adaptation of the approaches to reporting CRTs. First, SWTs randomly allocate clusters to groups that determine the timing of introduction of the intervention, rather than, as in CRTs, to the study control and intervention conditions [3]. There may be many groups to which clusters are allocated, and the number of groups will always be greater than the number of conditions. Second, in SWTs, the data corresponding to the intervention condition will be, on average, collected later than data corresponding to the control condition [3].

Participant flow, or CONSORT diagrams [6], are very often used in the reporting of CRTs [7]. These diagrams include rates of recruitment, refusal, drop-out, loss to follow-up, and missing outcome data by study condition among clusters and individuals [8, 9]. The large number of allocation groups and the crossover from control to intervention allocation can make it less straightforward to present a participant flow (that is, CONSORT diagram) for SWTs relative to CRTs.

Another almost universally supported characteristic of CRT reporting is an assessment of whether or not the randomisation procedure has resulted in study conditions that are balanced at baseline in terms of important covariates [6]. This is because although randomisation ensures that there is no systematic bias in allocation, the number of clusters may not be large enough to assume that there are no chance imbalances [10]. The large number of groups, and correspondingly small number of clusters per group, may mean that presentation of group characteristics is infeasible. Researchers may prefer to present an assessment of balance by condition. However, balance between the conditions of SWTs often also depends on the presence of secular trends in the outcome. To assess the risk of bias, it may be important to differentiate between imbalances that are due to chance and imbalances that are systematic.

The presence of a secular trend in the outcome will bias an unadjusted comparison between outcomes corresponding to intervention and control conditions [4]. Accounting for the potential bias from secular trends in the outcome is a key feature of the analysis methods (see below). Reporting and assessment of the trend is important for understanding the extent of the risk of bias, and the appropriateness of the analysis method.

### Analysis of an SWT

Ideally, the analysis method for a SWT will result in (1) an unbiased estimate of the intervention effect, (2) appropriately reflect the level of uncertainty in the point estimate, and (3) be as statistically efficient as possible.

Since SWTs are types of CRTs, principles of analysis for CRTs can be used to guide the analyses in SWTs. For instance, data on individuals, or any other sub-cluster unit, are likely to be correlated with data from others in the same cluster [11]. There is a rich literature on this issue because it also arises in parallel CRTs [10, 11]. However, analysis of SWTs poses some additional challenges. In particular, in SWTs the effect estimate is potentially confounded by secular changes in the outcome [4]. This is rarely an issue for CRTs as clusters allocated to the intervention and control conditions are usually followed up (that is, data are collected) over the same time period.

Taking these issues into consideration, there are several ways to analyse data from a SWT. These are primarily individual-level analyses and adopt one of two broad approaches to address potential bias from secular trends.

The first approach compares outcomes associated with the control and intervention conditions within the periods between successive crossover points, implicitly taking into account secular trends by conditioning on time. Observations corresponding to periods when all clusters are in the control or intervention condition do not contribute to the effect estimate (except indirectly to increase the precision by adjustment using outcome data from before randomisation). Parametric or semi-parametric models available include Cox regression or conditional logistic regression. Alternatively, researchers could calculate the intervention effect size for each of several time intervals, such as the periods between successive crossover points, and plot or summarise these. The advantage of this approach is that it preserves the randomization; it is sometimes referred to as a ‘vertical’ analysis [12]. This approach also avoids the need to specify time trends in the outcome. A disadvantage of a vertical analysis is that it is unclear how to acknowledge appropriately the clustering of participants over time within clusters. For this reason, we have not observed any strictly vertical analyses in the literature. While we have observed analysis by Cox regression conditioning on time [12], this was in conjunction with a random (frailty) effects analysis; so that, in order to account for clustering, the analysis used information over time and not solely vertical information in the estimate of the effect.

A second approach explicitly takes into account secular trends by producing an intervention effect adjusted for time trends, which are also estimated. This method compares outcomes corresponding to the control and intervention conditions within the periods between successive crossover points as well as between these periods in the same clusters, and maximises efficiency [4, 13]. This comparison includes, along with the vertical comparison, a controlled before-after comparison, sometimes referred to as a ‘horizontal comparison’, that is not, strictly speaking, a randomised comparison. The validity of this horizontal comparison therefore requires that the secular trend of the outcome be accounted for in each cluster. Secular trends may arise from changes in the level of the outcome in the population and also from changes in the constituents of the sample in the trial, for example, from attrition from a closed cohort. Time trends are commonly entered into the model as fixed effects, often as factors simply reflecting the periods between crossover points, with the assumption that the trend is the same in all clusters. This assumption may not be correct: the trend may vary across clusters and also may change in form when clusters cross over to the intervention condition. In some cases, the secular trend can be described using a linear trend (or higher orders) so as to reduce the number of parameters to be estimated; however, a companion paper in this series found that the number of parameters estimated does not substantially affect the power [13]. Researchers sometimes include outcome data in the dependent variable that was collected while all clusters are allocated to the either control or intervention conditions, which will introduce before-after comparisons that are not controlled and could introduce bias if the analysis model is badly mis-specified. This design decision is discussed in Copas *et al*. [3]

Individual-level models can gain efficiency and appropriately reflect the level of uncertainty in the point estimate reflecting the clustering in the data using random effects [4], generalized estimating equations (GEE) with a working correlation matrix (for example, exchangeable or autoregressive), or through robust standard errors. Multiple levels of clustering (for example, wards within hospitals or repeated measures of the same individuals) can be taken into account with these methods [14]. Adjustment for individual and cluster-level covariates can be made.

The standard mixed model approach to estimating the intervention effect, as described by Hussey and Hughes and ignoring further covariates for adjustment [4], involves fitting a model of the form:

$$ {Y}_{ijk}={\beta}_0+{\beta}_j+{\beta}_{effect}{X}_{ij}+{u}_i+{\varepsilon}_{ijk} $$

where the outcome *Y* is measured for individual *k* at time *j* within cluster *i*, *β*_*j*_ and *β*_*effect*_ are fixed effects for the *j* time points (often the periods between successive crossover points) and the intervention effect, respectively; *X*_ij_ is an indicator of whether cluster *i* has been allocated to start the intervention condition by time *j* (taking the value 0 if not and 1 if it has changed), and *u*_*i*_ is a cluster random effect with mean zero across clusters. The assumptions made by this model are not discussed in detail in Hussey and Hughes [4], and can be assessed. These include the lack of any interaction between the intervention and either time or duration of intervention exposure, and an assumption of exchangeability: that any two individuals are equally correlated within cluster regardless of whether in the same or different exposure conditions and regardless of time. A key further assumption is that the effect of the intervention is common across clusters. An important implication following from these assumptions - and the inclusion of comparisons of different periods between successive crossovers in the same clusters - is that, unlike in the typical CRT, much information concerning the population intervention effect can be gained from a small number of clusters if these have a large number of participants [4]. However, if the effect of the intervention is assumed to be, but is not, common across clusters, then the estimate of the intervention effect from the mixed effect model may have spuriously high precision. In mixed model analyses, varying intervention effects across clusters need to be explicitly considered, whereas the GEE approach is robust to mis-specifying the correlation of measurements within clusters, so it is less important to consider whether the effect varies across clusters in a GEE analysis.

### Lag in the intervention effect

Many interventions delivered at the cluster level will have a delay between the time when a cluster is allocated to start the intervention, and when changes in the outcome are likely to happen. This time is referred to here as the ‘lag-period’ and can be considered similar to a short-term ‘carry-over’ seen in one-way crossover trials [15]. In a SWT, lags may be due to training or installation time or because there is a lag in outcome response (for example, the delay in disease response to intervention). Although a lag in changes to the outcome in the intervention condition of a parallel trial may occur, it can be addressed by restricting measurement of outcomes, in both conditions, until after the lag-period is over. In SWTs, this is not so simple because the time between crossover points may not be long enough to avoid collecting data during the lag-period.

To account for hypothesised lags, investigators may consider including a fractional term for the intervention - that is, ranging from 0 to 1 - to reflect the time to reach full fidelity [16]. Alternatively, lags could be accounted for by excluding observations during the lag-period (similar to the ‘wash-out’ period in cross-over trials [17]), or shifting the crossover point so as to correspond with the end of the lag-period and assigning outcomes during the lag-period as corresponding to the control condition. Decisions about how to account for lags should be pre-specified so that they can be interpreted as ‘intention-to-treat’ analyses [18], as opposed to commonly conducted ‘on-treatment’ analyses, where being ‘on treatment’ is determined post hoc [19].

Ensuring fidelity of the intervention over time may be more challenging for an SWT than a CRT because many SWTs are conducted due to limitations in the capacity of the implementation set-up staff [20] and take place over long periods of time [1, 2]. Loss of fidelity may arise from the turnover of staff, degradation of equipment, or from an acquired ‘resistance’ to the intervention, for example, as would be expected with a behaviour-change advertisement campaign. This could be assessed analytically with an interaction between time since crossing over to intervention and the intervention effect (although this will have low power to detect a difference) or graphically.

Unlike for CRTs, no clear framework exists to guide when and how particular methods should be used that account for the challenging characteristics of SWTs described above. We therefore reviewed recently published SWTs to investigate the range of methods used by researchers to analyse and report these trials, appraise them, and make recommendations for future research.

## Methods

We systematically identified published SWT protocols and articles. The following sources were searched: PubMed, PsycINFO, CINAHL, Web of Knowledge, Cochrane Library, and the Current Controlled Trials Register on 14 May 2014. All English language papers published since 1 January 2010 that used a stepped wedge design were eligible. Studies that applied the stepped wedge method post hoc were excluded. The search returned studies with any of the following in the abstract: ‘stepped wedge’, ‘step wedge’, ‘experimentally staged introduction’, ‘delayed intervention’, or ‘one directional cross over design’. The results papers corresponding to protocols in the Mdege review were considered for inclusion [1]. Further details are found in the review paper in this series [2]. Two reviewers extracted data into a standardised form from all trials on the approaches to reporting and analysis used, with differences of opinion resolved through consultation. The risk of lag in the effect of the intervention or loss of fidelity over time was assessed subjectively from the description of the interventions, the timescale, the outcomes, and the context. We then selected and undertook a critical appraisal of three ‘case studies’. The three case studies were purposively selected to represent three designs used in different settings. These studies included many of the strengths and weaknesses that were found in the other studies reviewed. The purpose of this was to examine in more depth the approaches taken by these authors to report and analyse the studies.

This research was informed by published literature and therefore did not require ethical review.

## Results

We identified 10 articles published between 2010 and 2014 [2] (see Table 1).

**Table 1:** Reporting approaches used in the description of the trial results

Author, year	Diagram of SWT rollout	CONSORT-style diagram	Assessment of balance	Reported *k*/ICC	Group summaries between crossovers	Secular trend reported
Bacchieri, 2010 [29]	No	No	No	No	No	Yes, graph
Bashour, 2013 [22]	No	Yes	Yes: individual-level covariates at enrolment between conditions	Yes	No	Yes, fixed effects from model and a graph of the Likert responses by group
Durovni, 2013 [12]	No	Yes	No	Yes	No	No
Fuller, 2012 [23]	Yes	Yes	No	No	No	Yes, graphed modelled effect by condition
Gruber 2013 [21]	Yes	Yes	Yes: individual and household-level covariates at baseline by condition, weighted for time in condition	No	No	Yes, graphed by condition
Horner, 2012 [27]	Yes	No	Yes: cluster-level at baseline by group	No	Yes	Yes, model parameters, sub-groups, and graphs by group
Mhurchu, 2013 [24]	Yes	Yes	Yes: all schools described, with group indicated, but not summarised by group	No	Yes	Yes, using step-group summaries
Roy, 2013 [25]	Yes	Yes	Yes: cluster-level covariates and individual-level covariates at enrolment by condition	No	No	No
Schultz, 2014 [28]	Yes	No	Yes: individual-level at enrolment by condition	No	No	No
Stern, 2014 [26]	Yes	Yes	Yes: cluster-level covariates at baseline and individual-level covariates at enrolment by condition	No	No	No

Note: This table only includes information included in the main publication. Some articles included additional material in a supplement

### Reporting

Seven of the studies adapted the CONSORT diagram for CRTs [12, 21–26]. Three studies with closed-cohort designs adapted the CONSORT diagram to show the number of clusters and/or analysis units in each condition between successive crossovers [21, 22, 24]. Two studies with continuous recruitment of participants presented the number of participants followed up in each condition [12, 26]. The remaining two studies did not present either the length of follow-up in each condition or the number of participants contributing data from the control and intervention conditions [23, 25]. Seven studies presented a diagram of the rollout of the intervention [21, 23–28], and five studies– half of the studies – also included a CONSORT diagram [21, 23–26].

Seven of the studies reported an assessment of balance [21, 22, 24–28]. Of the three that did not, one was a closed-cohort design [29], and two were open-cohort designs [12, 23]. Of the seven that assessed balance, three reported balance by group at baseline [24, 26, 27]. Two of these assessed balance using cluster-level covariates only [26, 27]. The remaining study of the seven, Mhurchu *et al.*, reported baseline characteristics of all clusters and indicated which group they belonged to without summarising group-level statistics [24]. Five studies reported other assessments of balance by condition. Of these, one used a closed-cohort design and assessed balance between the conditions by weighting the baseline characteristics of each cluster and individual by the time spent allocated to each condition [21]. The remaining four reported balance between the conditions in terms of the characteristics of the participants when recruited since they used either an open-cohort design [6, 22, 26] or continuous recruitment [25].

Only two of the studies reported the extent of correlation between individuals in the same clusters [12, 22]. Only two reported simple summaries of the outcome in periods between successive crossovers [24, 27]; this was despite only three studies having more than 10 groups, beyond which it might be impractical to present the cluster summaries.

Six of the studies described the secular trend in the primary outcome [21, 22–24, 27, 29]. Five of these used a graph [21, 22, 23, 27, 29], with four of these presenting some disaggregation by condition [21, 23] or group [22, 27]. Only two of the nine studies that explicitly adjusted for time trends reported the secular trend parameter from the outcome analysis [22, 27].

### Analysis strategy

All studies analysed units at the sub-cluster level. In the majority of studies the units were individuals, but one study used households [21], another used pressure ulcers [26], and one used hand-hygiene opportunities [23]. Two studies collected data as time-to-event [12, 29], five collected repeated measures on individuals [21, 23, 24, 26, 27], and three collected single measures on individuals [22, 25, 28]. The measures of intervention effect included five odds ratios estimated using logistic regression [23–25, 27, 28]; one risk ratio estimated by log-Poisson regression (which also calculated risk difference using linear regression) [21]; two rate ratios, one estimated using Poisson regression [29] and the other using Cox regression [12]; and two mean differences estimated using linear regression [22, 26].

The correlation between observations on the same individuals (if relevant) and between individuals within the same cluster, was accounted for using random effects in the majority of cases, GEE in one case [21], and robust standard errors in two [21, 29]. One study did not account for correlation in the data within clusters due to convergence issues [28]. To account for potential confounding, eight of the studies adjusted their analysis for potentially confounding covariates [12, 21–27].

All studies used both vertical and horizontal information to estimate the effect of the intervention. Eight studies accounted for secular trends with a fixed effect term in the regression model, and one tested for interaction between the secular trend and intervention [26]. One did not account for secular trends [25]. Nine studies used data from before or after the rollout period in the outcome analysis. The remaining study analysed a time-to-event outcome by Cox regression, conditioning on time [12]. Since the model included a gamma-distributed random effect to account for within-cluster correlation, information from multiple periods between successive crossover points was used alongside the vertical analysis. One study [27] graphed the outcome over time by group and marked on the graph the time that the intervention was introduced, similar to a crude time-series approach.

In six studies, our reviewers judged that there was at least minor risk that the intervention effect would lag behind the introduction of the intervention (see Table 2). Only one study accounted for this in the analysis using fractional terms for intervention strength in a ‘per-protocol’ analysis [23]. In eight studies, reviewers believed that the effect may change over time because of either lapse in the fidelity of the delivery or desensitization of the population to the intervention’s effect.

**Table 2:** Primary outcome analysis methods

Author, year	Model fitted	Effect estimated	Vertical and/or horizontal analysis	Method to account for clustering	Repeated measures on individuals? Method used	Adjustment for confounding?	Adjustment for secular trends	Risk of lag	Accounting for lags	Risk of fidelity loss
Bacchieri, 2010 [29]	Poisson regression	Rate ratio	Both	Robust standard errors	Time-to-event	No	Fixed effect (no interaction)	None	None	Minor
Bashour, 2013 [22]	Linear regression	Mean difference	Both	Random effects	No	Yes	Fixed effect	None	None	Major
							(no interaction)			
Durovni, 2013 [12]	Cox regression	Rate ratio	Within-step only, plus frailty	Random effects	Time-to-event	Yes	Conditional	Major	None	Minor
Fuller, 2012 [23]	Logistic regression	Odds ratio	Both	Random effects	Yes, not explicitly accounted for	Yes	Fixed effects	Major	None (per-protocol analysis using observed lags)	Major
							(no interaction)			
Gruber 2013 [21]	Linear regression, Log-Poisson regression	Risk difference, risk ratio	Both	Robust standard errors, GEE	Yes, not explicitly accounted for	Yes, in secondary analyses	Fixed effect	Major	None	Low
							(no interaction)			
Horner, 2012 [27]	Logistic regression	Odds ratio	Both	Random effects	Yes, not explicitly accounted for	Yes	Fixed effect	Minor	None	Minor
							(no interaction)			
Mhurchu, 2013 [24]	Logistic regression	Odds ratio	Both	Random effects	Yes, pupil-level summaries and random effect	Yes	Fixed effect	None	None	Minor
							(no interaction)			
Roy, 2013 [25]	Logistic regression	Odds ratio	Both	Random effects	No	Yes	None	None	None	Minor
Schultz, 2014 [28]	Logistic regression	Odds ratio	Both	None: GEE not possible, and RE did not converge	No	No	Fixed effect	Minor	None	None
							(no interaction)			
Stern, 2014 [26]	Linear regression	Mean difference	Both	Random effects	Yes, multiple level random effects	Yes	Fixed effect with interaction	Major	None	Minor

### Case studies

#### Case study 1

Mhurchu *et al*. investigated the effect of providing free school breakfasts at school on pupils’ attendance [24]. The clusters were defined as schools. The design is described in Copas *et al*. [3]

In terms of reporting, a modified CONSORT diagram was included in the article showing participant flow in all four allocation groups. The article reported the number of pupils who withdrew or were lost over the school year in each group. Age, sex, and ethnicity at baseline were reported for the schools, but not attendance, for example, from previous term, and not aggregated by group. There was evidence from a table of attendance in each group that attendance reduced over the study period under both intervention and comparison conditions. There also appeared to be a decrease over time in the fidelity of the intervention after crossover (measured as the number of pupils actually attending breakfast). Time lags in intervention implementation or effect seemed unlikely to our reviewers, and no account was made for this in the analysis.

The primary outcome analysis model, inferred from the article, is shown below. Mhurchu *et al*. used logistic regression to model the log odds of good attendance, with a random intercept to account for clustering at school-level and another random effect to account for measurement of the same pupils within each school.

$$ log\left({Y}_{ijk}\right)=\mu +{\beta}_j+ ag{e}_{ik}+ gende{r}_{ik}+ ethnicit{y}_{ik}+\theta {X}_{ij}+{\alpha}_i+{\gamma}_{ik}+{e}_{ijk} $$

where

*Y*_*ijk*_ is the odds of poor attendance of pupil *k* over term *j* from school *i*;

*μ* is the log odds of poor attendance in the reference group (1st term in the control group with no free breakfasts);

*β*_*j*_ is a fixed effect adjusting for being in term *j*;

*age*_*ik*_, *gender*_*ik*_, and *ethnicity*_*ik*_ are fixed effects adjusting for the age, gender, and ethnicity of pupil *k* in school *i*;

*X*_*ij*_ is a fixed effect for whether or not school *i* has the intervention in term *j* (0 for no breakfasts, 1 for having free breakfasts);

*θ* is the log odds ratio for free school breakfasts on attendance;

*α*_*i*_ ~ *N*(0, *τ*^2^) is the random effect for clusters (schools);

*γ*_*ik*_ ~ *N*(0, *φ*^2^) is the random effect for each pupil; and

*e*_*ijk*_ ~ *N*(0, *σ*^2^) is the error term for each attendance record.

Given the design parameters, this analysis method seems appropriate. However, the inclusion of data corresponding to the final term where all pupils were in clusters allocated to receive breakfast constitutes an uncontrolled before-after comparison. The description of the participant flow could have been more detailed, for example by giving the number of schools as well as the number of pupils. Because participants experienced both the control and intervention phases, attrition may have affected the balance between the conditions: 12 % of participants withdrew or were lost to follow-up. Although the authors did describe the baseline characteristics of the schools, these were not aggregated by allocation group. Having only four time points limited the capacity to describe the time trend and the authors may have more efficiently captured secular trends with the day-level attendance records. The authors did not provide evidence that the secular trend was adequately captured by the fixed effect terms, for example, whether time trends changed on crossover to the intervention condition.

#### Case study 2

Durovni *et al*. (THRio study) [12] studied the effect of increased tuberculosis (TB) screening and preventive therapy on TB incidence among HIV clinic attendees, with clusters being the health clinic. The THRio study contained 29 clusters randomly allocated to 15 groups of 1-2, with a median time between successive crossover points of 2 months. Individual data were collected from an open cohort of HIV clinic attendees newly visiting these clinics over time. Outcome data were collected from patient records. A participant’s follow-up time corresponded to the control condition until after the participant visited a clinic that had initiated the intervention.

Durovni *et al*. included a simple participant flow diagram, giving the number of clusters in the trial and number of participants contributing to control and intervention periods. They did not report any losses to follow up. No information was provided on differences in important covariates between the 15 randomised groups or between the study conditions.

The primary outcome analysis model inferred from the article is shown below. Durovni *et al*. used Cox regression to model the hazard ratio for TB incidence and mortality, with calendar day being the underlying timeframe and a gamma-distributed random effect to account for clustering at the clinic level.

$$ {h}_{ik}(t)={h}_0(t) exp\left(\theta {X}_i(t)+{\alpha}_i+{e}_{ik}\right) $$

where

*h*_*ik*_(*t*) is the hazard of person *k* in clinic *i* being diagnosed with TB at time *t*;

*h*_0_(*t*) is the ‘baseline’ hazard of being diagnosed with TB at time *t* without the intervention, and is not specified;

*X*_*i*_(*t*) is a fixed effect for whether or not clinic *i* has the intervention at time *t*;

*θ* is the hazard ratio for the intervention on TB diagnoses;

*α*_*i*_ ~ *log gamma*(1, *τ*^2^) is the random effect for clusters (clinic); and

*e*_*ik*_ ~ *N*(0, *σ*^2^) is the error term for each person.

The authors used a model that was closely aligned to the manner of data collection. While Durovni *et al*. did not explore how the underlying trend in TB incidence was changing over the study period, the Cox analysis was conditional on the time of observation. However, whether or not the intervention effect changed over time, that is, the appropriateness of the proportional hazards assumption, was not shown. The primary analysis was unadjusted, but Durovni *et al*. also conducted a sensitivity analysis adjusting for age, gender, HIV antiretroviral therapy and CD4. Deaths or cases of TB within 60 days of the first clinic visit were excluded from the analysis; however, the authors did not account for any lag between control and intervention.

#### Case study 3

Fuller *et al*. investigated the effect of a hand-hygiene intervention for doctors and nurses on hand-hygiene compliance in hospitals [23]. The design is described in Copas *et al*. [3]

In terms of reporting, Fuller *et al*. provides a participant flow diagram, although several key elements are missing. The diagram is at a cluster level, and no information is given about the number of participants or which observations were conducted and when. It is also unclear whether or not there were any occasions on which visits to undertake hand-hygiene observation were not completed. No balance assessment table is present. A graphical assessment of the trend estimated by their primary analysis model showed a downward trend in compliance. This does not constitute a transparent presentation of the trends in the conditions, and appears not to present data from the post rollout period. The model did not include an interaction to assess whether the intervention effect changed over time.

The primary outcome model inferred from the article is given below. They used logistic regression with an interaction between ward type and intervention phase and two random effects for wards and hospitals to model the odds of good hand-hygiene.

$$ log\left({Y}_{imjk}\right)=\mu +{\beta}_j+ nstaf{f}_{im}+ staffrati{o}_{im}+\theta {X}_{imj}*typ{e}_{im}+{\alpha}_i+{\gamma}_{im}+{e}_{imjk} $$

where

*Y*_*imjk*_ is the odds of person *k* in hospital* i*, on ward *m*, complying with hand-hygiene at time* j*;

*μ* is the log odds of complying with hand-hygiene in the first observation;

*β*_*j*_ is a fixed effect adjusting for month *j*;

*nstaff*_*im*_, *staffratio*_*im*_ are fixed effects adjusting for number of staff, and the ratio of actual to expected staff in hospital *i*, ward *m*;

*X*_*imj*_ is a fixed effect for whether or not the hospital *i*, ward *m* had the intervention at time *j* (0 if observations were not being fed back to staff, 1 if they were);

*θ* is the log odds ratio for feeding back hand-hygiene compliance on hand-hygiene;

*type*_*im*_ is a fixed effect for the type of ward *m* in hospital *i*;

*α*_*i*_ ~ *N*(0, *τ*^2^) is the random effect for hospitals;

*γ*_*im*_ ~ *N*(0, *φ*^2^) is the random effect for ward m within hospital i; and

*e*_*imjk*_ ~ *N*(0, *σ*^2^) is the error term for each observation.

The analysis fitted a fixed effect term for the month of observations, and as a result, they included a large number of secular trend parameters. The analysis may have been more efficient if Fuller *et al*. had characterised the secular trend using a linear or other shaped trend, especially since they collected considerable data before and after the rollout period. Furthermore, in using considerable data from long periods both before and after the rollout period, the analysis appears to include a substantial degree of uncontrolled before-after comparison that may have biased the effect estimate or led to inappropriate precision as the assumptions of the analysis model need to be realistic throughout the period of data collection. The random effect for ward and hospital will have accounted for repeat measures of staff members and avoided imprecise random effects because of the small number of observations per staff member. Fuller *et al*. conducted a ‘per-protocol’ analysis with a time period corresponding to the observed delay in each cluster between allocation to the intervention and actual initiation of the intervention. This was intended to account for the lags in implementation, but does not correspond to a pre-hypothesised lag time as it was a post hoc ‘on treatment’ analysis based on the observed delay in implementation.

## Discussion

### Summary

We identified ten recent reports of SWTs. We found that several important aspects of SWTs were often not reported and that reporting practise was heterogeneous. While some of this heterogeneity arose from differences in the design of the studies, we conclude that standardised guidelines for reporting some of the more complex aspects of SWTs would be helpful. We offer some further ideas in this area below. Individual-level statistical models were used for the primary analysis of all included studies. Most of the models accounted for clustering in the outcome data for reported point estimates and associated confidence intervals. They also sought to adjust for secular trends in the outcome. This was usually done with a categorical variable corresponding to the periods between successive crossover points. Methods such as cubic splines and fractional polynomials could be useful to improve the estimations of time trends and, where data are sparse over time, would be more efficient. No studies explicitly anticipated potential time lags between intervention implementation and effect in the intention-to-treat analysis. No studies considered the possibility of different intervention effects across clusters.

### Reporting

The reporting of recent stepped wedge trials is heterogeneous and often inadequate. Only half of the studies reported both a diagram of rollout and a CONSORT style diagram and often with very little detail. This may be because of difficulty in adapting the CONSORT template, especially when the number of groups is large, although several studies failed to provide the details expected for reporting a CRT.

Only one study reported an assessment of balance using summaries of potential confounders by randomly allocated groups of clusters [27]. The others presented summaries by correspondence to control and intervention condition. In all but one of these studies, the potential confounders were measured in participants as they were enrolled into an open cohort and then summarised as corresponding to intervention or control depending on what condition the cluster was in at the point of enrolment. Any differences between the summaries of potential confounders corresponding to the two conditions may arise from the randomisation unsuccessfully balancing the groups at the start, or from changes in the participants who are enrolled over time. In other words: the summaries are affected by secular trends. Although not observed in our review, an analogous issue would arise for closed cohorts if the same method was applied (that is, summaries of potential confounders corresponding to the time when the clusters are in the intervention and control conditions). Differential attrition and time-varying confounders would result in summaries corresponding to each condition that differ because of trends as well as the allocation scheme. In contrast, the one exception - a closed-cohort study by Gruber *et al.* - summarised potential confounders measured at baseline, weighted by the time that each participant spent in clusters allocated to each condition [21]. This method assessed the extent of balance achieved between the conditions by the random allocation only - that is, without implicitly incorporating secular changes. This method can be applied only to variables available at baseline, for example, individual and cluster-level variables in closed-cohort designs and cluster-level variables only in open-cohort designs. Summaries by group, however, can incorporate data on participants enrolled throughout the trial since correspondence to any particular group is not time-dependent so long as enrolment is not affected by the intervention.

Summaries by group may help assess the likelihood that the randomisation successfully balanced expected outcomes, which is a requirement of the CONSORT statement [9]. As with CRTs, balance at baseline is the basis of the validity of vertical - that is, truly randomised - analyses. Summaries by group may be impractical when the number of clusters per group is small, and in such cases appraisal of the randomisation may be limited. Where possible, reporting both balance between groups and balance between conditions might be advisable so as to identify imbalances arising by chance as well as by secular changes.

As SWTs continue to be conducted and reported, further work will be required to advise how researchers should present data to assess imbalances due to the randomisation, as well as CONSORT diagrams, outcome frequencies, and balance between conditions. The appropriate adaptation of the CONSORT diagrams will depend on the design of the trial, in particular, whether participants are continuously recruited, exposed to more than one condition, and the number of crossover points.

### Analysis

All but one study used a method to account for clustered outcome data. A key question for analyses of SWTs is as follows: Has the secular trend been adequately adjusted for? The Cox regression used in Durovni *et al*. for a time-to-event is likely relatively robust to secular trends, so long as the model assumptions are met (for example, proportional hazards) because it conditions on time. Besides that approach, time trends were modelled explicitly. Few of the articles that we reviewed explored in detail how well modelling of secular trend was specified, all assumed that the trend was the same in all clusters, and only one assessed if it changed on crossover from control to intervention condition.

To assess whether a secular trend has been properly specified, we recommend presenting a graph of summary measures of the outcome in each condition in each of several appropriate time slices (for example, periods between successive crossover points), such as that used in Gruber et al. [21]. Confidence intervals or other measures of precisions should account for within-cluster correlation within the time slice. We recommend that particular care is taken to assess secular changes in the composition of the participants in a closed cohort and any attrition or censoring is reported clearly.

We found that only one of the studies tested for interaction between the intervention and calendar time or duration of intervention [26]. Potential interactions should be explored and reported. The interaction between the intervention effect and duration of the intervention could be explored as a primary parameter of interest itself. This possibility is a side-effect of the multiple data collection points and crossover points that are necessary in an SWT, a possibility that would not be available to a parallel CRT that did not have those features.

All of the studies except Durovni *et al*. included data from before and/or after the rollout period in the dependent variable. In the case of Fuller *et al.*, this appears to have constituted as much as 60 % of the data in the analysis [23]. Inclusion of data before and after the rollout period incorporates a before-after comparison that is uncontrolled, but is unlikely to lead to substantial bias if the effect of time is modelled in a flexible way. Data on the outcome from before the rollout period may be usefully used as a covariate to increase precision - for example, in an ANCOVA or logistic regression with baseline outcome data as a covariate - which is analogous to how these data are typically used in the analysis of CRTs, and could be applied to vertical analyses [10].

When the number of clusters is small, researchers need to be cautious with the use of random effects models to take into account between-cluster variability. Authors have advised against using such models in CRTs when there are small numbers of clusters per condition, with small being less than around 10-15 per condition [10, 11, 30]. GEE models with fewer than 40 clusters have been shown to be problematic [31], and Scott *et al*. have explored GEE methods suited to a modest numbers of clusters [32]. We believe that further work could examine the potential use of a cluster summary analysis approach, as recommended for CRTs with a modest number of clusters [10, 11]. None of the studies used a controlled time-series approach to analysis and further research could look into the potential gain from a richer analysis of longer-term trends in the data before rollout, where available. This approach may not circumvent many of the issues described previously; however, it has been shown to be effective in some circumstances [33].

All studies assumed that the intervention effect was the same in each cluster. However, in community trials in particular there is often a potential for the intervention to be implemented in a variety of different ways across clusters, indeed to be barely implemented at all in some clusters. In scenarios such as these, assuming a common effect of the intervention across clusters is inappropriate. This is crucial at the design stage (see Baio *et al*. [13]) because it suggests sample size calculation such as in Hussey and Hughes [4] may substantially underestimate the number of clusters required. It is also important not to report spurious precision in analysis, and when variation in intervention effect over clusters is likely, we strongly recommend methods that allow this are used such as GEE or mixed models with a random intervention by cluster term. An informal alternative may be to conduct an initial test of a common effect, and if this is not rejected, then apply a method that assumes a common effect.

We did not find an example of a strictly vertical analysis because even the example that used a Cox regression included a frailty to account for clustering. Identifying an efficient vertical analysis and comparing this to the results of mixed effects analysis in real SWTs is an important area for future research. Researchers looking for an analysis method that maintains the randomisation completely might consider calculating effects in each period between successive crossover points (using individual-level models accounting for clustering, or cluster summaries, as appropriate) and summarising these vertical analysis effects with a pre-specified method (for example, inverse variance weighting, or weighting by the balance between the number of clusters in the intervention and control conditions). To generate evidence against the null hypothesis, researchers can permute the allocation of clusters to groups according to the rules of the randomisation (for example, stratification), calculating an ‘effect’ of the intervention under each permutation, and locate the empirical effect estimate in the distribution of effects estimated under random chance. Permutation tests have been used in the analysis of CRT [34] and can be preferable to parametric methods [35]. A strictly vertical analysis is analogous to accepted methods for analysing CRTs. A limitation of a vertical approach is lower power relative to the mixed-effect or GEE model.

In this review, we have summarised and critiqued current practice in the reporting and analysis of SWTs. The review was limited to papers published after 2010, which may have reduced the capacity to observe emerging trends and innovations in analysis. The in-depth review of the case studies demonstrated the peculiarity of each case and identified idiosyncratic analysis and reporting elements, but these cannot be considered representative of all recent SWT reports. We have identified a number of questions for analysis and reporting of SWTs, and further work will be required to inform the literature.

## Conclusion

The reporting and analysis of recently conducted SWTs is varied. Substantial scope exists for improvement and standardisation in the reporting of trial parameters, including balance at baseline and attrition. We make recommendations for reporting in panels 1 and 2. The analysis of SWTs is often susceptible to bias if secular trends in the outcome are mis-specified in the analysis model. Trends, however, are rarely described in detail, and only a few reports of SWTs have cautiously explored the potential for bias. We make recommendations for how the analysis should be approached. Further research could explore the potential for bias with different analysis methods in more detail.

## Appendix

### Panel 1: Recommendations for reporting

**Table:**

Reporting recommendations Reports of SWTs should aim to achieve the following: 1. Include a diagram of study design with clusters, groups, and crossover points indicated on a calendar time-scale. 2. Include an adapted CONSORT diagram with cluster-level and individual-level participation and attrition. The adaptation should account for the follow-up time or the participant number corresponding to each condition, as appropriate to the design. 3. Unless impractical, include an assessment of balance between allocation groups on cluster-level characteristics at baseline and participant-level characteristics at recruitment. 4. Include an assessment of balance by condition, weighting any participants that contribute to both conditions by the time spent in each.	

### Panel 2: Recommendations for analysis

**Table:**

Analysis recommendations. Analyses of SWTs should aim to accomplish the following: 1. Use appropriate methods to adjust for clustering 2. Account for confounding from secular trends using an appropriate term for the trend in models for the outcome, and investigate potential effect modification of the intervention effect by time through including an interaction term. 3. Base the primary analysis of the intervention on data from the rollout period, together with data from exposure just before or after if collected. Data from just before the rollout period can be used for adjustment for differences at baseline. 4. Use Cox regression for time-to-event outcomes as this may be the more robust to secular trends. 5. Include a chart or table of outcome summaries by condition for each of several time intervals, to help check the form assumed for secular trends, and to investigate possible interaction between intervention and time. 6. Remember the assumptions made when applying mixed effect models and in particular consider whether it is appropriate to assume the intervention effect is common across all clusters.	

## Abbreviations

CRT: cluster randomised controlled trial
HIV: human immunodeficiency virus
SWT: stepped wedge cluster randomised controlled trial
TB: tuberculosis
